# Natural and Semisynthetic Analogues of Manadoperoxide B Reveal New Structural Requirements for Trypanocidal Activity

**DOI:** 10.3390/md11093297

**Published:** 2013-08-28

**Authors:** Giuseppina Chianese, Fernando Scala, Barbara Calcinai, Carlo Cerrano, Henny A. Dien, Marcel Kaiser, Deniz Tasdemir, Orazio Taglialatela-Scafati

**Affiliations:** 1 Department of Pharmacy, University of Naples “Federico II”, Via D. Montesano, 49, Naples I-80131, Italy; E-Mails: g.chianese @unina.it (G.C.); fernando.scala@unina.it (F.S.); 2 Department of Life and Environmental Sciences, Polytechnic University of Marche, Via Brecce Bianche, Ancona 60131, Italy; E-Mails: b.calcinai@univpm.it (B.C.); c.cerrano@univpm.it (C.C.); 3 Faculty of Fishery and Marine Science, Sam Ratulangi University, Manado 95115, Indonesia; E-Mail: hennydien@yahoo.com; 4 Department of Medical Parasitology and Infection Biology, Swiss Tropical and Public Health Institute, Basel CH-4002, Switzerland; E-Mail: marcel.kaiser@unibas.ch; 5 University of Basel, Petersplatz 1, Basel CH-4003, Switzerland; 6 School of Chemistry, National University of Ireland, Galway, University Road, Galway, Ireland; E-Mail: deniz.tasdemir@nuigalway.ie

**Keywords:** manadoperoxide B, marine antitrypanosomals, structure–activity relationships

## Abstract

Chemical analysis of the Indonesian sponge *Plakortis* cfr. *lita* afforded two new analogues of the potent trypanocidal agent manadoperoxide B (**1**), namely 12-isomanadoperoxide B (**2**) and manadoperoxidic acid B (**3**). These compounds were isolated along with a new short chain dicarboxylate monoester (**4**), bearing some interesting relationships with the polyketide endoperoxides found in this sponge. Some semi-synthetic analogues of manadoperoxide B (**6**–**8**) were prepared and evaluated for antitrypanosomal activity and cytotoxicity. These studies revealed crucial structure–activity relationships that should be taken into account in the design of optimized and simplified endoperoxyketal trypanocidal agents.

## 1. Introduction

Sleeping sickness (human African trypanosomiasis), a human disease caused by the single-celled protozoans *Trypanosoma brucei gambiense* (in Western and Central Africa) and *T. b. rhodesiense* (in Eastern and Southern Africa), is a devastating tropical disease and in 2012 the reported cases were over 7000 [1]. After transmission into humans by bites of *Glossina* flies, trypanosomes multiply in several tissues, including blood and lymph and, in a second stage, the immune response against the metabolites released causes the neurological symptoms, including behavioural changes, coma, and ultimately, if untreated, death. Disturbance of the sleep cycle, which gives the disease its name, is a characteristic feature of the cerebral stage of the disease.

The dramatic figures about spread and consequences of human African trypanosomiasis should be, at least partially, ascribed to the scarcity of efficacious, cheap and safe treatments. Eflornithine (in combination with nifurtimox) and the trivalent arsenic derivative melarsoprol are practically the only therapeutic options to treat the cerebral stage and their efficacy is reduced by the increasingly observed cases of cross-resistance [2]. Thus, there is an urgent need to find new, effective and, above all, affordable alternatives to the existing options for treatment of sleeping sickness.

**Figure 1:**
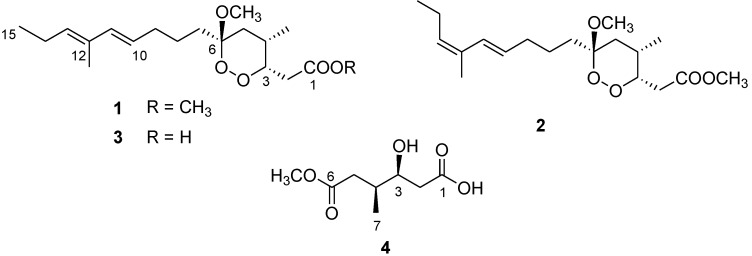
Chemical structure of manadoperoxide B (**1**) and of the new metabolites **2**–**4**.

In the course of our ongoing research investigation aimed at the discovery of marine secondary metabolites with potential activity against malaria and other tropical diseases [3,4,5], we have recently reported the isolation of manadoperoxide B (**1**) and its analogues manadoperoxides C–K from the sponge *Plakortis* cfr. *lita* de Laubenfels [6], a species widely distributed in the Indo-West Pacific, collected along the coasts of the Bunaken Marine Park of Manado (North Sulawesi, Indonesia). Some of these endoperoxyketal polyketides revealed a potent *in vitro* activity against *T. b. rhodesiense* and, remarkably, manadoperoxide B (**1**, Figure 1) proved to be an ultrapotent trypanocidal agent with an IC_50_ value of 3.0 ng/mL (8.8 nM), qualifying it as one of the most potent natural products, either marine or terrestrial, to possess such activity [6].

Structure–activity relationships within the series of isolated compounds disclosed the crucial role of substituents around the six-membered ring and, in particular of the methyl group attached at C-4 [6]. Surprisingly, when this methyl was linked at C-2 in place of C-4 (as found in peroxyplakoric ester B3) the activity was almost completely lost. As for the “western” side chain, the indications were not unambiguous. Also the non-dienic derivatives, such as manadoperoxides I and K, retained a very good activity with IC_50_ values 62 and 87 ng/mL, corresponding to 170 and 240 nM, respectively [6].

With this information in our hands, we have undertaken the chemical analysis of another specimen of *Plakortis* cfr. *lita* de Laubenfels, collected in the same area of the previous one, in order to obtain larger amounts of manadoperoxide B (**1**). During the fractionation of the organic extract of this sponge, we isolated two new analogues of 1, namely 12-isomanadoperoxide B (**2**) and manadoperoxidic acid B (**3**), along with a new dicarboxylate monoester derivative 4, whose structural elucidation is herein described (Figure 1). In this paper we also report on the preparation of three semisynthetic analogues of manadoperoxide B (**6**–**8**) and on the evaluation of the entire series of endoperoxyketal derivatives for *in vitro* trypanocidal activity against *T. b. rhodesiense*. 

## 2. Results and Discussion

### 2.1. Chemistry

The sponge, *Plakortis* cfr. *lita* de Laubenfels (order Homosclerophorida, family Plakinidae), was collected in January 2010 along the coasts of Bunaken Island (Manado, Indonesia). After homogenization, the organism was exhaustively extracted with MeOH and CHCl_3_. The combined extracts were subjected to MPLC chromatography over reversed-phase silica column and then selected fractions were purified by normal and reverse phase HPLC. Together with relatively large amounts of manadoperoxide B (**1**, approx. 0.23% of the organic extract), two new minor analogues, 12-isomanadoperoxide B (**2**, 0.023% of the organic extract) and manadoperoxidic acid B (**3**, 0.050% of the organic extract) were isolated.

Compound **2**, C_19_H_32_O_5_ by HR-ESIMS, was easily identified as a close analogue of **1**, differing only for modifications in the long “western” side chain. Accordingly, ^1^H and ^13^C NMR resonances (including proton multiplicities) for positions from C-1 to C-9 were practically superimposable to those detected for **1** [7], while consistent differences could be evidenced in the chemical shifts attributable to the diene system (H-10 from δ_H_ 5.47 in **1** to 5.60 in **2**; H-11 from δ_H_ 6.03 in **1** to 6.41 in **2**; H-13 from δ_H_ 5.26 in **1** to 5.40 in **2**). These differences could be ascribed to a configurational change around one or both the two double bonds. Since the *E* configuration at Δ^10^ was secured by the value of *J*_H-10/H-11_ (15.9 Hz), compound **2** should be the Δ^12^ isomer of **1**. This was unambiguously proved by the ROESY cross-peaks detected between H-11 and H_2_-14 and between 12-Me and H-13.

Analysis of most polar fractions of the crude extract afforded a further manadoperoxide B analogue, which was identified as the corresponding carboxylic acid (**3**). Manadoperoxidic acid B (**3**) showed HR-ESIMS data in agreement with the molecular formula C_18_H_30_O_5_, a methylene unit less than **1**. ^1^H and ^13^C NMR spectra of **3** were almost identical to those of **1**, with the single exception of the absence of the methyl ester signal in both spectra (δ_H_ 3.72, δ_C_ 52.2) plus the downfield shift of C-1 observed in the ^13^C NMR spectrum of **3** (δ_C_ 177.3 in **3**, instead of 172.5 in **1**). These data clearly indicated the presence of a carboxylic acid at C-1 in place of the methyl ester group. This was finally proved by treatment of a small aliquot of **3** with diazomethane, which gave manadoperoxide B (identified by NMR and [α]_D_) and in a quantitative yield.

**Figure 2:**
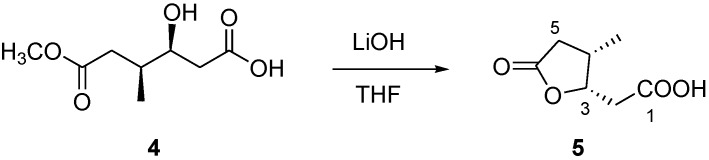
Conversion of compound **4** into the lactone **5**.

During the purification procedure of **3**, we also obtained small amounts of the dicarboxylic acid monoester **4**, C_8_H_14_O_5_ by HR-ESIMS. ^1^H NMR spectrum of **4** showed a methyl doublet signal at δ_H_ 0.95, a series of signals between δ_H_ 2.11 and 2.52, a methyl singlet at δ_H_ 3.66 and an oxymethine at δ_H_ 4.04. Inspection of the COSY spectrum allowed us to arrange all the multiplets of this spectrum within the same spin system going from C-2 to C-5 and including the oxymethine at C-3 and a methyl branching at C-4. All the proton signals were connected to those of the directly linked carbon atoms by means of the HSQC spectrum, while the HMBC cross-peaks between H-2/C-1, H_2_-5/C-6 and 6-OMe/C-6 allowed the correct location of the terminal carboxylic acid and ester functionalities, thus defining the planar structure of **4**. In order to establish the relative configuration of the two stereogenic centers C-3 and C-4, we reasoned that a transformation of the ester group at C-6 into the corresponding carboxylic acid would have led to the formation of the γ-lactone, particularly useful to solve this stereochemical problem. Thus, minute amounts of compound **4** were treated with LiOH in THF/H_2_O at 0 °C to obtain the lactone **5** in good yield (Figure 2). After complete assignment of NMR data of compound **5**, the ROESY cross-peaks H_2_-2/4-Me clearly indicated the *cis* relationship between the two substituents on the lactone ring, thus disclosing the relative configuration also for compound **4**.

**Figure 3:**
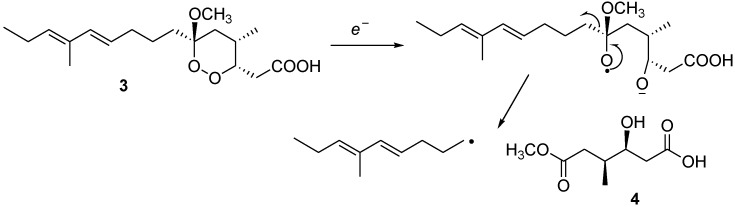
Postulated origin of compound **4** from manadoperoxidic acid B (**3**).

The isolation of compound **4** from this sponge is particularly interesting taking into account its evident structural relationships with the C-1/C-6 moiety of manadoperoxides. Figure 3 illustrates a plausible derivation of compound **4** from manadoperoxidic acid B: a single electron reduction of the endoperoxide bond would afford an oxygen radical at C-6, which should then rearrange to give cleavage of the C-6/C-7 bond, thus forming compound **4** and an alkyl radical.

Intriguingly, several years ago we have reported the isolation of simplactones from a Caribbean specimen of *Plakortis simplex*, an organism particularly rich of the antimalarial endoperoxide plakortin [8]. We can hypothesize that simplactones could have with plakortin a very similar relationship as that illustrated for compound **4** and manadoperoxides in Figure 3. Of course, we cannot exclude the alternative possibility that compound **4** and simplactones do not derive from the corresponding endoperoxide derivatives but they are the biogenetic precursors. Since the biosynthesis of plakortin and other bioactive endoperoxides is the subject of intense investigations [9], this second hypothesis would be worthy of being further explored.

**Figure 4:**
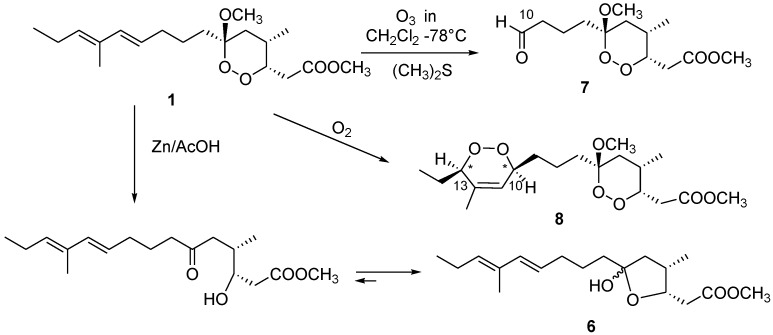
Semisynthetic transformations on manadoperoxide B (**1**).

The availability of consistent amounts of manadoperoxide B (**1**) from the sponge material gave us the opportunity to prepare some semi-synthetic derivatives of **1**, in order to increase the chemical diversity for evaluation of antitrypanosomal activity. In particular, three derivatives **6**–**8** (Figure 4) have been prepared. Briefly, reductive cleavage (Zn/AcOH) of the endoperoxide bond of **1** yielded the diastereomeric mixture of hemiketals **6** [7]. Ozonolysis of **1** with reductive work-up afforded the new aldehyde **7**, whose ^1^H NMR spectrum completely lacked double bond signals, whilst the aldehyde proton signal was evident at δ_H_ 9.78. Finally, a solution of **1** and the photo-sensitizer methylene blue in chloroform was irradiated under an oxygen atmosphere with a halogen lamp (500W) for 24 h at −20 °C to obtain the photo-oxygenation reaction, affording the new endoperoxide derivative **8** as a mixture of the two diastereomers showing *cis* orientation of the substituents at C-10 and C-13. Also in this case, the structure and stereochemistry of the obtained product(s), expected on the basis of the described mechanism of the reaction [10], was secured by 1D and 2D NMR analysis.

### 2.2. Trypanocidal Activity

Natural and semi-synthetic analogues of manadoperoxide B (**2**, **3**, **6**–**8**) were evaluated *in vitro* for their antitrypanosomal activity against bloodstream forms of *Trypanosoma brucei rhodesiense.* As shown in Table 1, 12-isomanadoperoxide B (**2**) was the most potent compound, with the closest IC_50_ value (11 ng/mL) to that of manadoperoxide B (**1**, IC_50_ 3 ng/mL). It was followed by the semi-synthetic derivative **8** (IC_50_ 0.16 μg/mL) and the equipotent manadoperoxidic acid B (**3**) and compound **7** with low μg/mL level efficacy. The compound **6** was inactive at the highest test concentrations (20 μg/mL). The same compounds were also tested for cytotoxicity against L6 cells (a primary cell line derived from rat skeletal myoblasts) and they generally exhibited moderate to low cytotoxic activity. Among the newly tested compounds, 12-isomanadoperoxide B (**2**) appeared to display the largest selectivity index (SI, calculated by dividing the IC_50_ value against L6 cells to the IC_50_ value against the parasite) of 345. This is however 10 times lower than that of manadoperoxide B (**1**) (Table 1).

**Table 1:** *In vitro* antiprotozoal (IC_50_) and cytotoxic activity (IC_50_) of **2**,** 3**,** 6**–**8** *^a^*.

Compounds	*T. b. rhodesiense*	Cytotoxicity L6 cells
Manadoperoxide B (**1**)	0.003 *^b^* (0.0088)	10.8 *^c^* (31.76)
12-Isomanadoperoxide B (**2**)	0.011 (0.032)	3.80 (11.18)
Manadoperoxidic acid B (**3**)	1.87 (5.74)	7.12 (21.84)
Compound **6**	>20	82.30 (252.4)
Aldehyde **7**	1.21 (4.42)	7.55 (27.55)
Compound **8**	0.16 (0.43)	12.58 (33.82)
Melarsoprol	0.002 (0.0050)	7.3 (18.25)
Podophyllotoxin	--------	0.004 (0.0096)

*^a^* IC_50_ values are in μg/mL (in µM in parentheses) and mean values from at least two replicates which varied ≤ ±50%; *^b^* Data from ref. [6]; *^c^* Against HMEC-1 cell line, from ref. [6].

These results draw further structure–activity relationships to those previously determined for this class of compounds [6]. The complete inactivity of compound **6**, sharing with **1** the entire carbon skeleton and differing only for the presence of the lactol ring in place of the endoperoxide, clearly shows the crucial role of this latter functionality for the trypanocidal activity. The modest activity exhibited by the truncated aldehyde **7** highlights the importance of the 10,12-diene system, although compound **8**, in which this system is converted into a dioxin ring, retains a significant activity. The change in the geometry at Δ^12^ of the diene, experienced by compound **2**, causes four-fold reduction of the activity of manadoperoxide B (**1**). Most remarkably, compound **3**, the carboxylic acid derivative of **1**, is about one thousand times less active, most likely due to the increase of polarity, although it is not clear at this stage whether this is a pharmacokinetic or a pharmacodynamic effect. However, this information is very precious for the future design of optimized manadoperoxide B derivatives, since it clearly suggests the need for a non-hydrolysable functionality at C-1, thus trying to minimize the possible *in vivo* conversion of **1** into the much less active **3**. Non-hydrolysable lipophilic derivatives should also be able to better penetrate through the blood brain barrier (BBB), which is critical for the treatment of cerebral stage of *Trypanosoma* infections.

## 3. Experimental Section

### 3.1. General Experimental Procedures

Low and high resolution ESI-MS spectra were performed on a LTQ OrbitrapXL (Thermo Scientific) mass spectrometer. ^1^H (700 MHz) and ^13^C (175 MHz) NMR spectra were measured on Varian INOVA spectrometers. Chemical shifts were referenced to the residual solvent signal (CDCl_3_: δ_H_ 7.26, δ_C_ 77.0; CD_3_OD: δ_H_ 3.34). Homonuclear ^1^H connectivities were determined by the COSY experiment. Through-space ^1^H connectivities were evidenced using a ROESY experiment with a mixing time of 500 ms. One-bond heteronuclear ^1^H-^13^C connectivities was determined by the HSQC experiment; two- and three-bond ^1^H-^13^C connectivities by gradient-HMBC experiments optimized for a ^2,3^*J* of 8 Hz. Medium pressure liquid chromatography was performed on a Büchi apparatus using a reverse-phase (230–400 mesh) column. HPLC were achieved on a Knauer apparatus equipped with a refractive index detector and LUNA (Phenomenex) SI60 or Kinetex (2.6 µ, 100 × 4.60 mm Phenomenex) C18 columns.

### 3.2. Animal Material, Extraction, Isolation

A specimen of *Plakortis lita* de Laubenfels (order Homosclerophorida, family Plakinidae) was collected in January 2010 along the coasts of the Bunaken Island in the Bunaken Marine Park of Manado. A frozen voucher sample (Man/10/02-02) has been deposited at the Dipartimento di Farmacia, Università di Napoli Federico II. After homogenization, the organism was exhaustively extracted, in sequence, with methanol and chloroform. The combined organic extracts (9.08 g) were subjected to MPLC chromatography over C18 silica column (200–400 mesh) eluting with a solvent gradient of decreasing polarity from water to MeOH (H_2_O/MeOH 9:1; H_2_O/MeOH 8:2 and so on with progressive 10% increase of MeOH to reach 100% MeOH) to chloroform (MeOH followed by MeOH/CHCl_3_ 1:1 and then CHCl_3_). Fractions eluted with H_2_O/MeOH (1:9) were combined and further fractionated by gravity column chromatography on silica gel using a *n*-hexane/EtOAc gradient (*n*-hex/EtOAc 9:1; *n*-hex/EtOAc 8:2; *n*-hex/EtOAc 7:3 and then EtOAc). Fractions eluted with *n*-hexane/EtOAc (9:1) mixture were subjected to repeated column and HPLC chromatographies (*n*-hexane/EtOAc 96:4) affording manadoperoxides B (**1**, 20.5 mg) and 12-isomanadoperoxide B (**2**, 2.1 mg). Fractions of the RP-MPLC column eluted with H_2_O/MeOH 4:6 were re-chromatographed by RP-HPLC (MeOH/H_2_O 6:4, flow 0.8 mL/min) affording manadoperoxidic acid B (**3**, 7.3 mg) and compound **4** (2.1 mg).

### 3.3. 12-Isomanadoperoxide B (**2**)

Colorless amorphous solid; [α]^25^_D_ −7.5 (*c* 0.1 in CHCl_3_); ^1^H NMR (CDCl_3_, 500 MHz) δ_H_ 6.41 (1H, d, *J* = 15.9 Hz, H-11), 5.60 (1H, dt, *J* = 15.9, 6.0 Hz, H-10), 5.26 (1H, t, *J* = 6.1 Hz, H-13), 4.43 (1H, m, H-3), 3.74 (3H, s, 1-OMe), 3.26 (3H, s, 6-OMe), 2.97 (1H, dd, *J* = 15.5, 9.5 Hz, H-2a), 2.57 (1H, m, H-4), 2.44 (1H, dd, *J* = 15.5, 4.5 Hz, H-2b), 2.14 (2H, overlapped, H-14), 2.10 (2H, overlapped, H-9), 1.79 (3H, s, 12-Me), 1.69 (1H, overlapped, H-5a), 1.66 (1H, overlapped, H-7a), 1.40 (2H, overlapped, H-8), 1.36 (1H, overlapped, H-7b), 1.28 (1H, m, H-5b), 0.98 (3H, t, *J* = 7.1 Hz, H-15), 0.84 (3H, d, *J* = 7.1 Hz, 4-Me); ^13^C NMR (CDCl_3_, 125 MHz) δ_C_ 172.5 (C, C-1), 138.5 (CH, C-11), 136.8 (CH, C-13), 133.9 (C, C-12), 126.4 (CH, C-10), 103.2 (C, C-6), 80.12 (CH, C-3), 52.0(CH_3_, 1-OMe), 48.8 (CH_3_, 6-OMe), 34.6 (CH_2_, C-5), 33.9 (CH_2_, C-9), 32.1 (CH_2_, C-7), 31.4 (CH_2_, C-2), 27.6 (CH, C-4), 23.8 (CH_2_, C-8), 22.7 (CH_2_, C-14), 17.0 (CH_3_, C-4-Me), 15.0 (CH_3_, C-12-Me), 13.4 (CH_3_, C-15); (+) ESI-MS *m/z* 341 [M + H]^+^, 363 [M + Na]^+^. HR-ESIMS *m/z* 341.2325 (calcd for C_19_H_33_O_5_ 341.2328).

### 3.4. Manadoperoxidic Acid B (**3**)

Colorless solid; [α]^25^_D_ −5.0 (*c* 0.2 in CHCl_3_); ^1^H NMR (CDCl_3_, 500 MHz) δ_H_ 6.03 (1H, d, *J* = 15.9 Hz, H-11), 5.47 (1H, dt, *J* = 15.9, 6.0 Hz, H-10), 5.40 (1H, t, *J* = 6.1 Hz, H-13), 4.46 (1H, m, H-3), 3.26 (3H, s, 6-OMe), 2.95 (1H, dd, *J* = 15.5, 9.5 Hz, H-2a), 2.57 (1H, m, H-4), 2.47 (1H, dd, *J* = 15.5, 4.5 Hz, H-2b), 2.09 (2H, overlapped, H-14), 2.07 (2H, overlapped, H-9), 1.70 (3H, s, 12-Me), 1.69 (1H, overlapped, H-5a), 1.66 (1H, overlapped, H-7a), 1.40 (2H, overlapped, H-8), 1.36 (1H, overlapped, H-7b), 1.28 (1H, m, H-5b), 1.00 (3H, t, *J* = 7.1 Hz, H-15), 0.84 (3H, d, *J* = 7.1 Hz, 4-Me); ^13^C NMR (CDCl_3_, 125 MHz) δ_C_ 177.3 (C, C-1), 136.0 (CH, C-11), 133.8 (CH, C-13), 133.3 (C, C-13), 125.8 (CH, C-10), 103.4 (C, C-6), 80.2 (CH, C-3), 52.2 (CH_3_, 1-OMe), 48.8 (CH_3_, 6-OMe), 34.6 (CH_2_, C-5), 33.6 (CH_2_, C-9), 32.1 (CH_2_, C-7), 31.4 (CH_2_, C-2), 27.6 (CH, C-4), 23.5 (CH_2_, C-8), 21.9 (CH_2_, C-14), 17.0 (CH_3_, C-4-Me), 15.0 (CH_3_, C-12-Me), 13.0 (CH_3_, C-15); (−) ESI-MS *m/z* 325 [M − H]^−^. HR-ESIMS *m/z* 325.2013 (calcd for C_18_H_29_O_5_ 325.2015).

### 3.5. Diazomethane Reaction of Manadoperoxidic Acid B (**3**)

Manadoperoxidic acid B (**3**, 1.0 mg) was dissolved in ethyl ether and the resulting solution was added dropwise to an ethereal solution of CH_2_N_2_ (*ca.* 15 equiv) at 0 °C. The mixture was stirred for 10 min and then concentrated under reduce pressure to give semisynthetic manadoperoxide B (**1**) identified by means of NMR and [α]^25^_D_.

### 3.6. Compound **4**

Colorless solid; [α]^25^_D_ −23.0 (*c* 0.1 in CHCl_3_); ^1^H NMR (CDCl_3_, 500 MHz) δ_H_ 4.04, (1H, m, H-3), 3.66 (3H, s, 6-OMe), 2.97 (1H, bs, 3-OH), 2.52 (1H, dd, *J* = 12.2, 6.0 Hz, H-5a), 2.48 (1H, overlapped, H-2a), 2.43 (1H, overlapped, H-2b), 2.23 (1H, dd, *J* = 12.2, 4.5 Hz, H-5b), 2.11 (1H, m, H-4), 0.95 (3H, d, *J* = 7.1 Hz, 4-Me); (^13^C NMR (CDCl_3_, 125 MHz) δ_C_ 177.8 (C, C-1), 173.6 (C, C-6), 70.2 (CH, C-3), 52.7 (CH_3_, 6-OMe), 37.7 (CH_2_, C-5) 35.0 (CH, C-4), 31.8 (CH_2_, C-2), 17.4 (CH_3_, C-4-Me); (−) ESI-MS *m/z* 189 [M − H]^−^. HR-ESIMS *m/z* 189.0770 (calcd for C_8_H_13_O_5_ 189.0763).

### 3.7. Conversion of Compound **4** into Lactone **5**

Compound **4** (1.1 mg) was dissolved in a THF/H_2_O 3:1 solution (2.0 mL) and 2 mg of LiOH were added. The solution was stirred at 0 °C overnight. Then, the reaction mixture was partitioned between EtOAc and water. The organic phase, evaporated to dryness, contained compound pure compound **5** (0.6 mg).

### 3.8. Compound **5**

Colorless solid; [α]^25^_D_ −11.0 (*c* 0.1 in CHCl_3_); ^1^H NMR (CD_3_OD, 500 MHz) δ_H_ 4.97 (1H, m, H-3), 2.80 (1H, dd, *J* = 16.7, 7.5 Hz, H-5a), 2.77 (1H, m, H-4), 2.59 (1H, dd, *J* = 12.2, 4.5 Hz, H-2a), 2.43 (1H, dd, *J* = 12.2, 6.0 Hz, H-2b), 2.20 (1H, dd, *J* = 16.7, 3.3 Hz, H-5b), 1.07 (3H, d, *J* = 7.1 Hz, 4-Me);^13^C NMR (CDCl_3_, 125 MHz) δ_C_ 176.6 (C, C-1), 176.0 (C, C-6), 82.2 (CH, C-3), 37.1 (CH, C-4), 36.8 (CH_2_, C-2), 32.9 (CH_2_, C-5), 14.0 (CH_3_, C-4-Me); (−) ESI-MS *m/z* 157 [M − H]^−^. HR-ESIMS *m/z* 157.0507 (calcd for C_7_H_9_O_4_ 157.0501).

### 3.9. Reductive Cleavage of Manadoperoxide B

Semi-synthetic procedures and spectral data of **6** are reported in ref. [7].

### 3.10. Reductive Ozonolysis of Manadoperoxide B

A stream of O_3_ was bubbled into a solution of manadoperoxide B (**1**, 4.1 mg, 0.012 mm) in CH_2_Cl_2_ (2 mL) kept at −78 °C until a blue-colored solution resulted. After stirring for 1 min, excess of O_3_ was removed upon bubbling N_2_ and dry Me_2_S (1 mL) was added to the colorless solution. The reaction mixture was left at room temperature overnight, then concentrated *in vacuo*, purified by reversed-phase HPLC (eluent MeOH/H_2_O 55:45) to yield compound **7** (1.1 mg) in the pure state.

### 3.11. Aldehyde **7**

Colorless amorphous solid. [α]^25^_D_ −3.5 (*c* 0.1 in CHCl_3_); ^1^H NMR (CDCl_3_): δ_H_ 9.78 (1H, bs, H-10), 4.43 (1H, ddd, *J* = 9.5, 4.3, 3.0 Hz, H-3), 3.72 (3H, s, 1-OMe), 3.26 (3H, s, 6-OMe), 2.97 (1H, dd, *J* = 15.5, 9.5 Hz, H-2a), 2.57 (1H, m, H-4), 2.44 (1H, overlapped, H-2b), 2.41 (2H, overlapped, H_2_-9), 1.69 (1H, overlapped, H-5a), 1.71 (1H, overlapped, H-7a), 1.67 (2H, overlapped, H_2_-8), 1.62 (1H, overlapped, H-5b), 1.34 (1H, m, H-7b), 0.86 (3H, d, *J* = 7.1 Hz, 4-Me); ESIMS: *m/z* 297 [M + Na]^+^, HRESIMS: *m/z* 297.1307, calcd. for C_13_H_22_O_6_Na *m/*z 297.1314.

### 3.12. Photo-Oxygenation Reaction

A solution of manadoperoxide B (**1**, 9.0 mg) in CHCl_3_/MeOH 95:5 (2 mL) was photolysed with a 500 W halogen lamp in the presence of methylene blue (0.01 mg) as photosensitizer, through which was bubbled a constant stream of oxygen at a flow rate of 50 mL/min for 24 h. The reaction was performed in a Pyrex flask fitted with an external cooling jacket. The reaction mixture was then concentrated *in vacuo* and the resulting residue was purified by HPLC chromatography (*n-*hexane/EtOAc mixtures 85:15) to yield pure compound **8** (3.0 mg).

### 3.13. Compound **8**

Colorless oil. [α]^25^_D_ −2.5 (*c* 0.1 in CHCl_3_); ^1^H NMR (CDCl_3_): δ 5.65 (1H, bs, H-11), 4.45 (1H, m, H-3), 4.30 (1H, overlapped, H-13), 4.29 (1H, overlapped, H-10), 3.72 (3H, s, 1-OCH_3_), 3.27 (3H, s, 6-OCH_3_); 2.92 (1H, dd, *J =*15.5, 9.4 Hz, H-2a), 2.56 (1H, m, H-4), 2.44 (1H, dd, *J =*15.5, 3.6 Hz, H-2b), 1.75 (1H, overlapped, H-9a), 1.72 (3H, s, 12-Me), 1.70 (1H, overlapped, H-5a), 1.69 (1H, overlapped, H-7a), 1.58 (2H, overlapped, H_2_-14), 1.55 (1H, overlapped, H-5b); 1.32 (1H, overlapped, H-7b), 1.25 (2H, overlapped, H_2_-8), 1.00 (3H, t, *J =*7 Hz, H_3_-15), 0.85 (3H, d, *J =*7 Hz, 4-Me). ESI-MS: *m/z* 395 [M + Na]^+^, HR-ESIMS: *m/z* 395.2051, calcd. for C_19_H_32_O_7_Na *m/*z 395.2046.

### 3.14. Activity against *Trypanosoma brucei rhodesiense*

Minimum Essential Medium (50 µL) supplemented with 25 mM HEPES, 1 g/L additional glucose, 1% MEM non-essential amino acids (100×), 0.2 mM 2-mercaptoethanol, 1mM Na-pyruvate and 15% heat inactivated horse serum was added to each well of a 96-well microtiter plate. Serial drug dilutions of eleven 3-fold dilution steps covering a range from 100 to 0.002 μg/mL were prepared. Then 4 × 10^4^ bloodstream forms of STIB 900 strain (the stock was isolated in 1982 from a human patient in Tanzania and after several mouse passages was cloned and adapted to axenic culture conditions) [11] of *T. b. rhodesiense* in 50 μL was added to each well and the plate incubated at 37 °C under a 5% CO_2_ atmosphere for 72 h. 10 µL of a resazurin solution (prepared dissolving 12.5 mg resazurin in 100 mL double distilled water) [12] was then added to each well and incubation continued for a further 2–4 h. Then the plates were read in a Spectramax Gemini XS microplate fluorometer (Molecular Devices Cooperation, Sunnyvale, CA, USA) using an excitation wavelength of 536 nm and an emission wavelength of 588 nm. The IC_50_ values were calculated by linear regression [13] from the sigmoidal dose inhibition curves using SoftmaxPro software (Molecular Devices Cooperation, Sunnyvale, CA, USA). Melarsoprol was the standard drug.

### 3.15. Cytotoxicity against L6-Cells

Assays were performed in 96-well microtiter plates, each well containing 100 μL of RPMI 1640 medium supplemented with 1% L-glutamine (200 mM) and 10% fetal bovine serum, and 4 × 10^4^ L-6 cells. Serial drug dilutions of eleven 3-fold dilution steps covering a range from 100 to 0.002 μg/mL were prepared. After 72 h of incubation the plates were inspected under an inverted microscope to assure growth of the controls and sterile conditions. 10 μL of a resazurin solution (prepared dissolving 12.5 mg resazurin in 100 mL double distilled water) was then added to each well and the plates incubated for another 2 h. The plates were read with a Spectramax Gemini XS microplate fluorometer using an excitation wavelength of 536 nm and an emission wavelength of 588 nm. The IC_50_ values were calculated by linear regression [13] from the sigmoidal dose inhibition curves using SoftmaxPro software (Molecular Devices Cooperation, Sunnyvale, CA, USA). The reported IC_50_ values are the means of at least two separate experiments. Podophyllotoxin was used as control drug.

## 4. Conclusions

In this paper we have described the isolation of two simple analogues of the ultrapotent trypanocidal agent manadoperoxide B (**1**), namely the carboxylic acid (**3**) and the 12 *Z*-derivatives (**2**). These compounds, along with the semi-synthetic derivatives **6**–**8**, have been tested against *T. brucei rhodesiense* allowing a useful extension of the available structure–activity relationships. In particular, our data supports the 1,2-dioxane ring to be the key pharmacophore, but also points out the importance of the side chain diene system for the activity; although some derivatives showing a modified version of this group still have significant activity. In our previous investigation on manadoperoxides B–K [6] we already showed the deleterious effect of an increase in the polarity of the side chain; we have now verified that a decrease in lipophilicity due to modifications at C-1 also causes dramatic effects in trypanocidal potency. The data now available on structure–activity relationships will be taken into account in the design of optimized and simplified endoperoxyketal trypanocidal agents.
